# Topologically robust sound propagation in an angular-momentum-biased graphene-like resonator lattice

**DOI:** 10.1038/ncomms9260

**Published:** 2015-10-06

**Authors:** Alexander B. Khanikaev, Romain Fleury, S. Hossein Mousavi, Andrea Alù

**Affiliations:** 1 Department of Physics, Queens College of The City University of New York, Queens, New York 11367, USA; 2 Department of Physics, The Graduate Center of The City University of New York, New York, New York 10016, USA; 3 The Department of Electrical and Computer Engineering, The University of Texas at Austin, 1616 Guadalupe Street, UTA 7.215, Austin, Texas 78701, USA

## Abstract

Topological insulators do not allow conduction in the bulk, yet they support edge modes that travel along the boundary only in one direction, determined by the carried electron spin, with inherent robustness to defects and disorder. Topological insulators have inspired analogues in photonics and optics, in which one-way edge propagation in topologically protected two-dimensional materials is achieved breaking time-reversal symmetry with a magnetic bias. Here, we introduce the concept of topological order in classical acoustics, realizing robust topological protection and one-way edge propagation of sound in a suitably designed resonator lattice biased with angular momentum, forming the acoustic analogue of a magnetically biased graphene layer. Extending the concept of an acoustic nonreciprocal circulator based on angular-momentum bias, time-reversal symmetry is broken here using moderate rotational motion of air within each element of the lattice, which takes the role of the electron spin in determining the direction of modal edge propagation.

## Abstract (web summary)

Topological order for sound remains largely unexplored. Here, Khanikaev *et al*. introduce the concept of topological order in classical acoustics, realizing robust topological protection and one-way edge propagation of sound in a suitably designed resonator lattice, thus expanding the ability to tailor acoustic waves.

## Graphical abstract

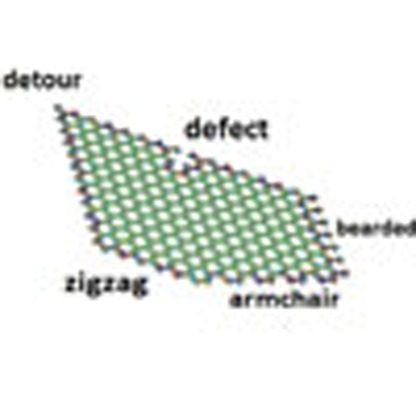

The concept of topological order, originally discovered in condensed matter physics^1^,^2^,^3^,^4^,^5^,^6^,^7^,^8^,^9^,^10^, has recently inspired scientists working in many branches of physics and engineering to look for topologically nontrivial states in several fields of interest. Topological states have been discovered in two-dimensional and three-dimensional materials, at the basis of the quantum Hall effect (QHE), quantum spin Hall effect and topological insulators. These concepts have also inspired photonic analogues, such as photonic crystals^11^,^12^,^13^,^14^,^15^,^16^, arrays of silicon-ring resonators^17^,^18^, bianisotropic metamaterials^19^ and chiral waveguides^20^,^21^, opening exciting new directions in optics. The inherent robustness against local defects^15^ and disorder^22^, provided by the topological nature of these phenomena, has allowed overcoming common rules of wave scattering and interference in topological insulators and their analogues. As an example, the edge states supported by these structures can seamlessly flow around sharp bends and defects, avoiding backscattering^15^,^17^,^18^,^19^,^20^ and inspiring interesting functionalities for topologically protected optical components.

In condensed matter, topological states of matter are inherently related to time-reversal symmetry. In the particular case of QHE^1^,^10^, as well as for its photonic analogue realized in magnetically biased photonic crystals^8^,^9^,^10^,^11^,^16^, time-reversal symmetry is suitably broken to realize one-way nonreciprocal edge modal dispersion^19^. In this case, propagation is allowed only in one particular propagation direction, making backscattering impossible. In fermionic systems with time-reversal symmetry, a form of topological protection can still arise. This is due to Kramer's theorem, which ensures the existence of time-reversal partner edge states with their electron spin being locked to the propagation direction, and therefore no backscattering can occur in absence of spin-flip processes^23^,^24^. In bosonic and classical systems, the latter class of protection is not available, but some restricted protection can be still achieved based on spatial or internal symmetries (for example, duality in electromagnetics), provided that a conserved pseudo-spin, odd under time-reversal symmetry, may be judiciously engineered^19^. Genuine topological protection for bosons and classical waves, however, is only possible in nonreciprocal systems with broken time-reversal symmetry. Since acoustic waves do not significantly interact with an external magnetic bias, topological order for sound has not been explored to date. In this paper, on the contrary, we show that these recent advances in quantum physics may be extended to acoustic systems using angular-momentum bias, dramatically expanding our ability to tailor acoustic waves.

While throughout the past centuries we have mastered the manipulation of sound propagation and scattering, perfecting musical instruments, music halls and whispering galleries, it is still challenging to break the inherent symmetry with which sound travels in space. Nonreciprocal acoustic response in magneto-elastic materials has been explored in ref. 25, but no experimental confirmation of large magnetic-based nonreciprocity or isolation has been demonstrated to date, due to the inherently weak coupling between magnetic and acoustic effects. An alternative approach to break time-reversal symmetry and achieve nonreciprocity has been recently suggested^26^. It has been shown that the effects of a magnetostatic bias may be replaced by the application of angular momentum, or rotational motion, in suitably designed acoustic resonators, leading to nonreciprocal response and giant isolation, and providing the foundations for a new class of nonreciprocal acoustic devices—acoustic circulators. On the basis of this discovery, here we introduce a new approach to topological order in periodic acoustic systems biased with angular momentum, where time-reversal symmetry is broken by rotational motion. We apply these concepts to demonstrate that topologically nontrivial states with strong robustness can be obtained in acoustic lattices with broken time-reversal symmetry.

More specifically, here we study an acoustic system mimicking a magnetically biased graphene lattice, as schematically shown in Fig. 1a. A graphene layer constitutes a well-established platform to realize topological order in condensed matter systems^27^. Its hexagonal lattice supports a Dirac point whose inherent time-reversal symmetry may be broken, for example, by an applied magnetic bias, to reveal Landau levels separated by bandgaps that support topologically protected edge states^28^,^29^. In our acoustic analogue geometry, the graphene-like lattice is formed by a planar periodic array of subwavelength acoustic resonators interconnected by hollow tubes to form a hexagonal lattice^30^. The resonators are formed by two hard-walled coaxial cylinders, with the inner space filled by air. As the air starts flowing, with moderate velocity as discussed in the following, the imparted angular momentum can break time-reversal symmetry, and realize the analogue of a magnetically biased graphene layer for sound.

## Results

### Infinite diatomic lattice

**Figure 1:**
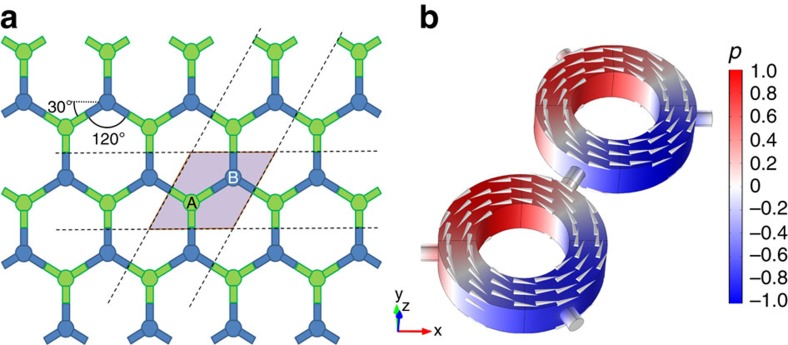
Diatomic lattice forming an acoustic analogue of graphene. (**a**) Lattice with two rotated Y-junctions (A and B, respectively) per unit cell (shaded region). (**b**) One unit cell of the lattice modelled in COMSOL Multiphysics, with acoustic pressure distribution shown in colour for one of the Dirac modes of interest. The grey arrows indicate the direction of airflow in the resonators. Structure dimensions are: inner and outer radius of the cavity are *R*_in_=5.08 cm and *R*_out_=9.21 cm, respectively, height of the cavity *H*=4.45 cm.

When disconnected from the lattice, each acoustic resonator in Fig. 1a supports two, clockwise and counterclockwise, lowest order modes, with no modulation in the vertical *z*-direction, and with eigenfrequencies *ω*_+/−_ corresponding to *l*=±1 angular momentum^26^. If the medium inside the resonators is stationary, these modes are degenerate, that is, *ω*_+_=*ω*_−_; however, as soon as an angular momentum bias in the form of air rotation is applied to the resonators, the degeneracy is lifted by the amount Δ*ω*=*ω*_+_−*ω*_−_=*v*_air_/*D*, where *v*_air_ is the fluid velocity and *D* is a parameter associated with the resonator geometry^26^.

When the resonators are connected in the hexagonal lattice of Fig. 1a, the clockwise and counterclockwise modes couple, forming a complex acoustic band structure. We apply a first-principle approach based on the direct solution of the equations of sound propagation in moving media to find modes and band structure for this system, as detailed in Methods. In parallel, we also developed an analytical model based on coupled-mode theory and the scattering matrix formalism^31^, outlined in the Methods section, which agrees well with the full-wave modelling performed with COMSOL Multiphysics. While all calculations presented in this section are obtained under the assumption of a constant air velocity inside the resonators, we have also performed studies in which airflow exchange between coupled resonators is fully considered. As described in the Methods section, for this case we performed full-wave multiphysics simulations with acoustic equations solved in conjunction with stationary Navier–Stokes equations for fluid flow, assuming that the air is brought to motion by a fan inserted inside every resonator, producing a pressure difference across its boundaries. The velocity fields calculated using this first-principle approach has an effect identical to the simplified uniform filed profile assumed in this section (see Methods for details), serving also as a confirmation for the inherent robustness of the topological effects described in the following.

**Figure 2:**
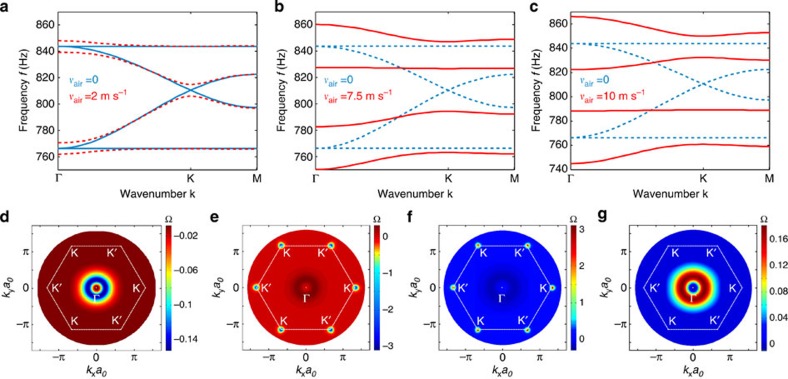
Bulk band structure with different levels of airflow. (**a**–**c**) Band diagrams for bulk acoustic modes obtained using first-principle numerical calculations based on finite element method. (**a**) The blue solid lines show the case of the unbiased lattice, supporting a conventional Dirac point in the spectrum. Red dashed lines correspond to the case of slow air rotation inside the resonators. (**b**,**c**) Red solid lines show the band diagrams for special values of fluid velocity, inducing a vanishing group velocity for former top and bottom Dirac bands, respectively. For reference, the blue dashed lines refer to the stationary case, consistent with **a**. (**d**–**g**) Berry curvatures of the 1st, 2nd, 3rd and 4th bands, respectively, for angular momentum bias *v*_air_=2 m s^−1^.

Figure 2a–c shows the band structure, calculated based on finite-element simulations (and validated with coupled-mode theory in Supplementary Figs 1–4), for the unit cell shown in Fig. 1b, with air velocity gradually increasing in each resonator, as indicated in each panel. The band diagram is calculated around the frequency of 800 Hz, for which four dipolar *l*=±1 acoustic bands may be observed. Due to the hexagonal symmetry of the lattice, in the stationary case (panel a, blue solid lines, time-reversal symmetry is preserved) the band diagram has a Dirac-like linear dispersion, with double degeneracy located at the K-point (*k*_y_=0, *k*_x_=4*π*/3*a*_0_), analogous to wave propagation along a graphene layer. As an example, the pressure field profile corresponding to the lower Dirac band near the K-point in a unit cell is shown in Fig. 1b. In addition to the two fast Dirac bands, we also observe two slow modes that do not propagate in the lattice. Note that, since the same changes in the band structure due to the air motion take place near the K′ point of the Brillouin zone (*k*_y_=0, *k*_x_=−4*π*/3*a*_0_), the band structure at K′ is identical to the one at the K-point and, therefore, it is omitted from the figures.

After applying a nonzero angular momentum in the resonators (red dashed lines in panel a), as shown by the grey arrows in Fig. 1b, time-reversal symmetry is broken and the Dirac degeneracy is lifted. Being induced by time-reversal symmetry breaking, the produced gap has topological nature and a topological index—the Chern number—can be assigned to the bulk acoustic bands^12^,^14^,^32^. To confirm the topologically nontrivial nature of the crystal, we calculated the Berry curvatures for all four bands of interest, using field profiles obtained from first-principle finite-element simulations, as shown in Fig. 2d–g. The Berry curvature was subsequently integrated over the entire Brillouin zone to obtain the Chern numbers of the bands. For the case of clockwise fluid rotation, the Chern number assumed values *C*={−1,0,0,1} for the four bands shown in Fig.2a–c, confirming their topologically nontrivial nature. When the sense of rotation in the resonators is reversed to counterclockwise, the sign of the topological indexes also reverses, and the Chern number assumes values *C*={1,0,0,−1}. Fig. 2d,g confirm that the nonvanishing Chern number for the first and fourth bands emerges entirely from the removal of their degeneracies with the second and third bands at the Γ-point. For the second and third bands, in contrast, Fig. 2e,f shows that both degeneracies (first and fourth bands at the Γ point, and second and third bands at K and K′ points) contribute opposite values (+1 and −1) and (−1 and +1), respectively, leading to a vanishing net value of the Chern number.

Of particular interest are the cases with air rotation velocities *v*_air_=7.5 m s^−1^ and *v*_air_=10 m s^−1^ (Fig. 2b,c), for which the band dispersion has interesting analogies with QHE. In these cases, the group velocity vanishes in the two Dirac bands, second and third, respectively. This is analogous to the case of a two-dimensional electron gas in the presence of a magnetic field bias, when Landau levels with vanishing group velocity emerge. Thus, in addition to opening a topological gap, the rotational bias may inhibit propagation of acoustic waves as bulk modes. It is interesting that the values of critical velocities required here to realize such frozen sound states do not coincide with those for which ideal isolation is achieved in the single acoustic resonator case, as considered in ref. 26. This may be understood as the effect of constructive wave interference within the lattice, required to form closed Landau-like orbits.

### Topological edge states

The appearance of topologically robust edge modes is the most fascinating manifestation of topologically nontrivial states. In the acoustic system under study we consider two different types of edge states: (i) acoustic modes that appear on the external edges of the system, and (ii) modes confined to domain walls formed by a reversal of the applied angular momentum inside the structure. According to the bulk-boundary correspondence principle^31^, the number of edge states found at a particular interface is determined by the change in the sum of the Chern numbers of all the bulk bands of lower frequency across the interface^15^. Thus, for the first class of edge modes we expect only one mode for any crystal termination.

**Figure 3:**
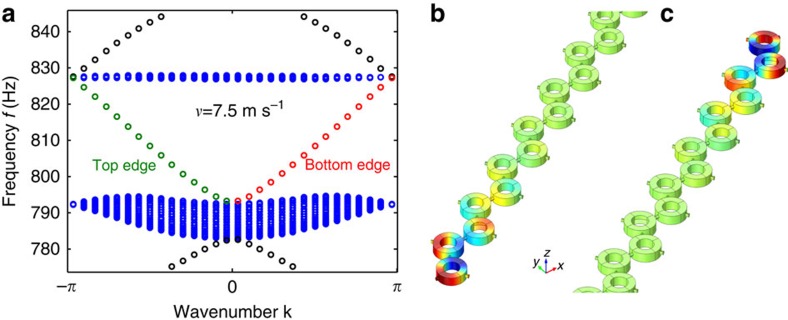
Topological edge modes in acoustic graphene. (**a**) Acoustic band structure for a supercell of 20 unit cells and a uniform rotational bias velocity *v*_air_=7.5 m s^−1^. Bulk modes are shown by blue and edge modes by black, green and red coloured markers. (**b**,**c**) Acoustic pressure profiles of the one-way edge mode localized at the bottom and top of the supercell, respectively, corresponding to the red and green bands in **a**.

Figure 3a shows a representative example of the band structure in such a case, for a 1 × 20 supercell with *v*_air_=7.5 m s^−1^. The figure reveals two edge bands within the acoustic band gap. Inspection of the pressure profiles, shown in Fig. 3b,c, confirms that these bands correspond to modes localized on the top and bottom edges of the system, respectively. These modes have one-way character and transfer energy only in the forward (backward) direction for the bottom (top) edge mode, having respectively positive and negative group velocity *v*_g_. It is interesting that the direction of energy flow is dictated by the direction of airflow at the edges of the crystal, peculiarly similar to the way electron spin controls the direction of edge propagation in conventional topological insulators.

**Figure 4:**
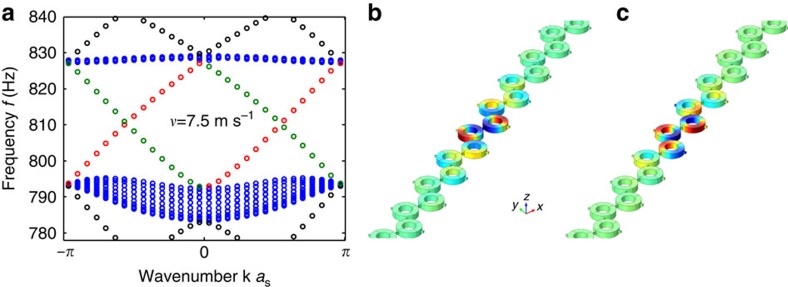
Topological edge modes confined to a domain wall. (**a**) Acoustic band structure for a supercell of 20 unit cells with domain wall at the centre. The angular momentum bias is flipped within the lattice on one specific boundary along the domain wall from *v*=7.5 m s^−1^ to=−7.5 m s^−1^. Bulk modes are shown in blue, and edge modes in black, green and red coloured markers, respectively. Modes associated with red bands are localized in the middle of the supercell, as shown in **b** and **c**, while the green bands are localized at the external edges of the supercell (not shown). (**b**,**c**) Acoustic pressure profiles of the one-way edge modes localized at the domain wall with positive and negative phase velocities, respectively, corresponding to the two red bands in **a**.

Fig. 4 considers the case of a domain wall, that is, a sudden reversal of rotation velocity taking place within the lattice. According to the bulk-boundary correspondence principle, the difference in the band Chern numbers between the two domains equals two in this scenario, and therefore two edge modes should be supported by the domain wall. Due to the way our simulation model is set up, with periodic boundary conditions on the top and bottom boundaries of the supercell, a second domain wall appears, leading to a total of four edge modes within the bang gap region (shown by red and green circles in Fig. 4a), with two modes localized at every wall. Fig. 4b shows the acoustic pressure profiles of the two modes localized at the central domain wall, which correspond to red bands in Fig. 4a, and are formed by symmetric and anti-symmetric bonding of the evanescent waves supported at the edge in the two domains. As seen in the band diagram, these two modes have again one-way character, and can transfer energy only in the forward, but not backward direction. The direction of energy transfer reverses with a change of air rotation in the resonators, which is observed for modes shown by green lines in Fig. 4a confined to the domain wall at the external edges (pressure field profiles not shown).

## Discussion

**Figure 5:**
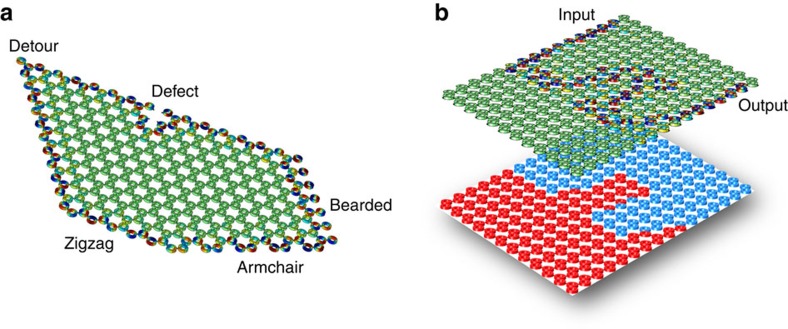
Topological robustness of acoustic edge modes. (**a**) A one-way (counterclockwise) edge mode propagates along different cuts of the acoustic graphene lattice, and flows around deliberately introduced defect (top zigzag cut) without backscattering and formation of standing-wave patterns. (**b**) Excitation of a one-way (counterclockwise) edge mode and its propagation (top subplot) along an irregularly shaped domain wall created by the reversal of the Doppler bias (bottom subplot).

To confirm the topological robustness of the acoustic edge modes described in the previous figures, we have performed large-scale simulations of acoustic graphene lattices in which we deliberately introduced defects of different kinds. For any nontopological-guided edge mode, we would expect strong reflection at sharp corners or defects, and the formation of standing-wave patterns along the walls due to interference effects. However, for the case of one-way topological edge modes, as described in this paper, we clearly observe strong robustness against such structural defects, as seen in Fig. 5a. Here we consider a plethora of possible defects and boundary variations: the edge mode seamlessly detours between zigzag, armchair and bearded edges of the finite crystal. In the same figure, we have also confirmed the robustness of the edge modes against local defects introduced by removing several resonators from the edge of the lattice.

In addition to robustness, the edge states of the considered domain wall allow ideal reflection-less routing along arbitrarily defined pathways, reconfigurable in real time by simply creating line boundaries within the lattice with opposite applied angular momenta on the two sides. Indeed, the path of the edge mode can be dynamically reconfigured by reshaping the domain wall through the change in rotation direction of the fluid inside the resonators. To confirm the possibility of such topologically robust routing, we have generated an irregularly shaped domain wall with velocity bias map shown in the lower subplot of Fig. 5b. As seen in the upper panel of Fig. 5b, the edge mode excited at the top edge of the crystal, after travelling along the edge, enters the bulk at the domain wall. Inside the crystal, the edge mode travels along the path defined by the domain wall and leaves the bulk of the crystal at the opposite edge without any back-reflection, confirming the possibility of dynamically controllable reflection-less routing of sound in a topologically protected lattice.

To conclude, we have introduced here the concept of topological order in acoustic metamaterials, obtained by properly controlling the applied angular momentum in suitably engineered resonator lattices. Extending advanced quantum physics concepts to the realms of classical acoustics, we have envisioned unprecedented possibilities to route and manipulate sound, achieving unusual propagation effects. We believe that topological acoustic concepts can dramatically expand the engineering toolkit of modern acoustic devices, and bring forward a new versatile way to control sound waves. Topological robustness and the ability to guide sound waves around arbitrarily shaped pathways, as demonstrated here, represents just the start of a plethora of fascinating phenomena stemming from the topological nature of angular-momentum-biased acoustic systems. During the review process, a somewhat related proposal for topological acoustics has been presented in ref. 33. Due to the open geometry and nonresonant nature of the proposed lattice elements, we argue that such alternative realization of a topological acoustic lattice would require significantly faster fluid rotation to achieve noticeable topological gap opening.

## Methods

### Coupled-mode theory for an acoustic cavity under angular-momentum bias

The temporal coupled-mode equations for the amplitudes *a*_+_ and *a*_−_ of the right-handed and left-handed modes of an azimuthally symmetric acoustic cavity under angular-momentum bias read^26^:


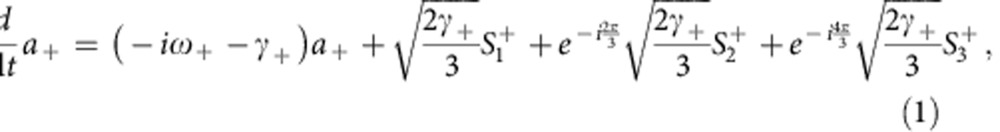



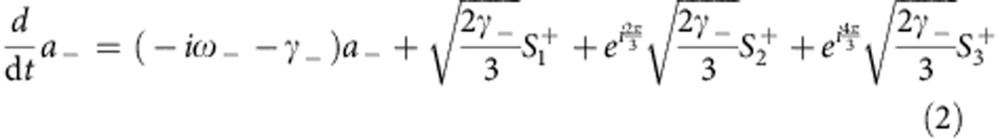


where *ω*_±_ are the eigenfrequencies of the right and left-handed modes, and *γ*_±_ is the inverse of their decay times to the output channels 1, 2 and 3, placed at the azimuthal positions *ϕ*=0, 2*π*/3 and 4*π*/3, respectively. In equation (1), we have also included the excitation signals at ports *i*, 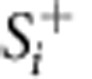. The output signals 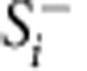 at the three ports are due to the interference between the direct reflection and the fields leaking from each mode,


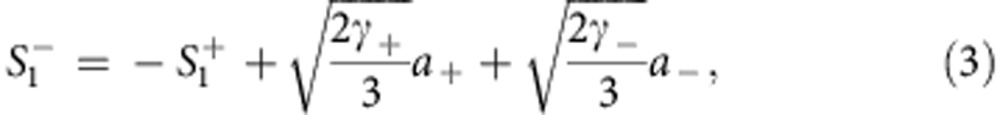



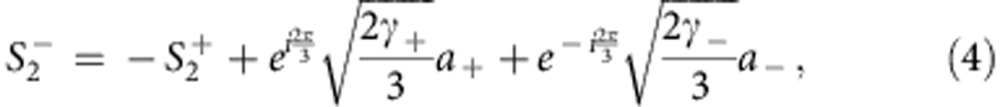



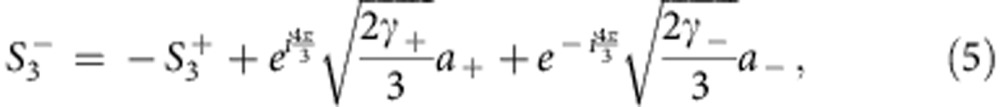


Next we assume harmonic excitation, at port 1 only 
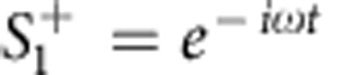
, 
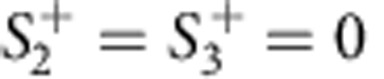
. After the variable change *a*_±_=*a*_±_*e*^−1*ωt*^, we obtain the following system of equations:


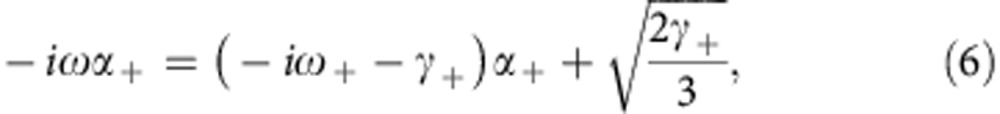



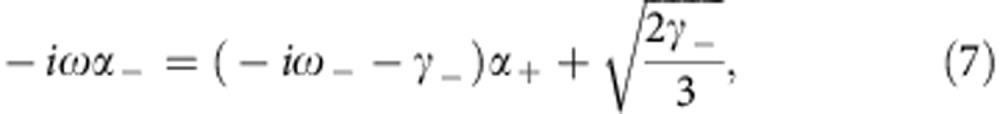


whose solution is


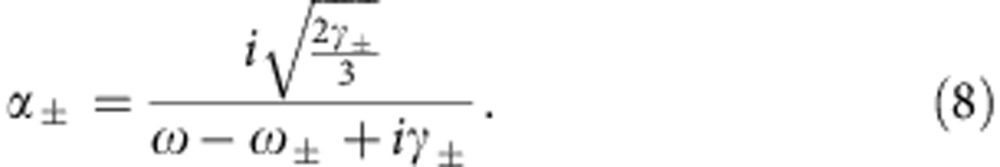


The scattering parameters 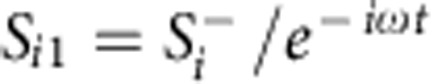 are then directly obtained from equations (3, 4), yielding


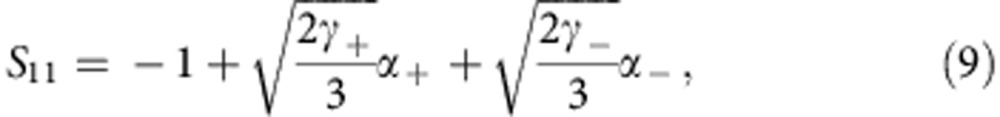



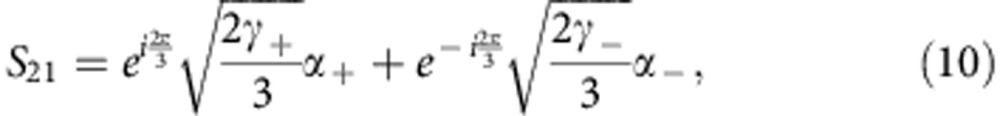



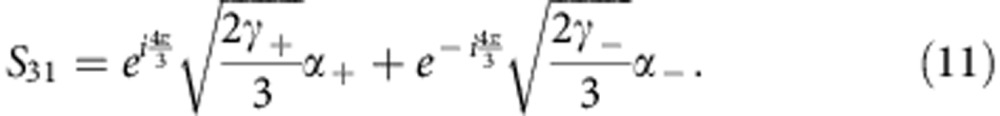


By symmetry, we deduce the full scattering matrix using the following equations:


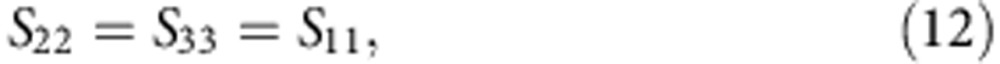



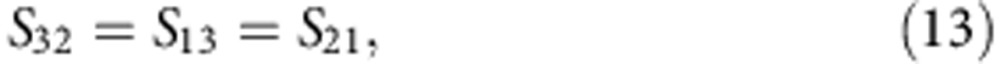



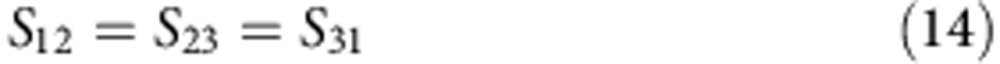


For our case of angular-momentum bias, the resonance frequencies are given by *ω*_±_=*ω*_0_±*v*/*R*_av_, where *R*_av_ is the average radius of the cavity^26^. The decay constants are obtained by fitting the full-wave simulations. Supplementary Fig. 1 shows the evolution of the scattering spectrum of a single ring cavity as the angular-momentum bias is gradually increased.

### Scattering matrix of one unit cell

The scattering matrix of the unit cell of the acoustic graphene layer (Supplementary Fig. 2), consisting of two ring cavities, may be calculated from the scattering matrix of the single cavity from equations (9, 10, 11, 12, 13, 14). As shown in the figure, we call 1, 2 and 5 the ports of the first circulator A and 3, 4 and 6 the ones of the second. The output signals *b*_*i*_ at the ports 
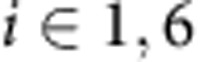
 are related to the input signals *a*_*i*_ by the individual circulator scattering elements *s*_*ij*_


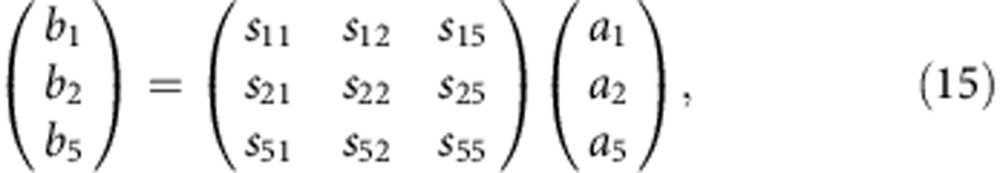


and


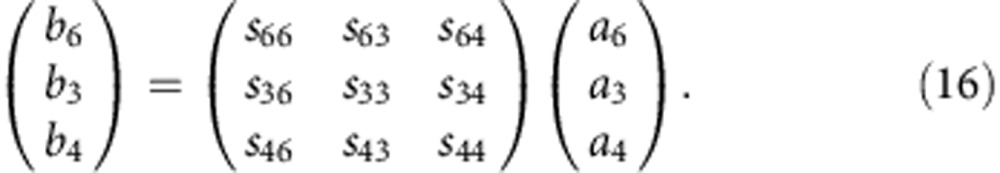


To obtain the scattering matrix 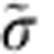 of the 4-port unit cell, we use the additional equations *a*_6_=*b*_5_ and *a*_5_=*b*_6_ and obtain, after some straightforward algebra


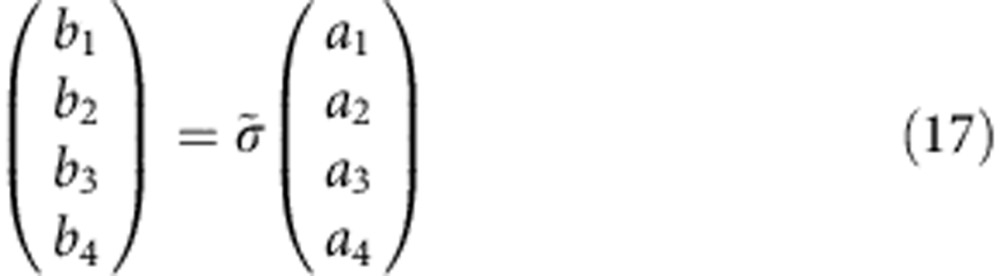


with


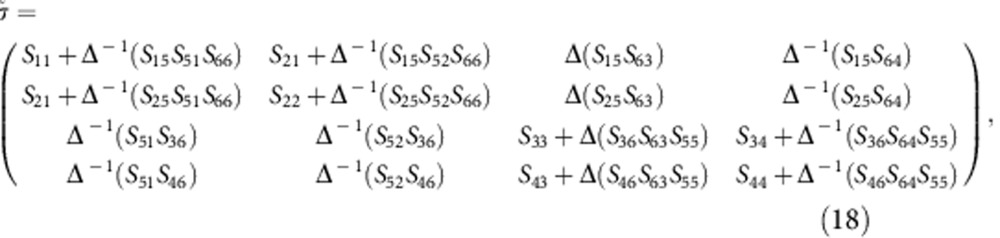



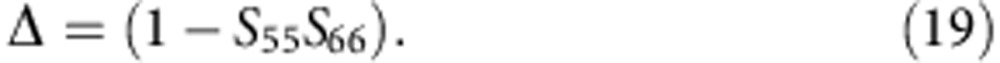


### Analytically derived bulk band structure

To obtain the bulk band structure, we apply Bloch theorem to both input and output signals of the 4 × 4 unit cell, obtaining


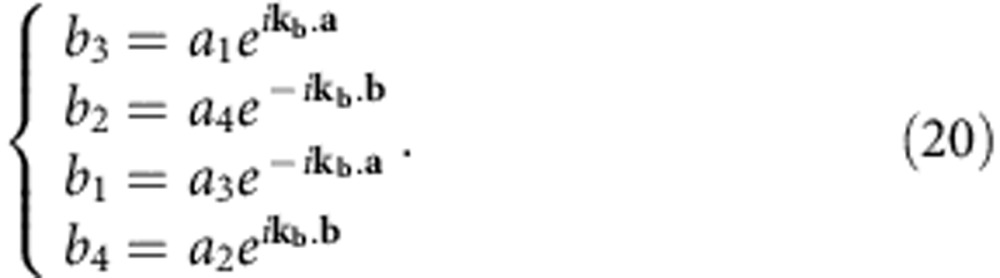


We can write the system equation in matrix form, introducing the Bloch matrix 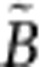:


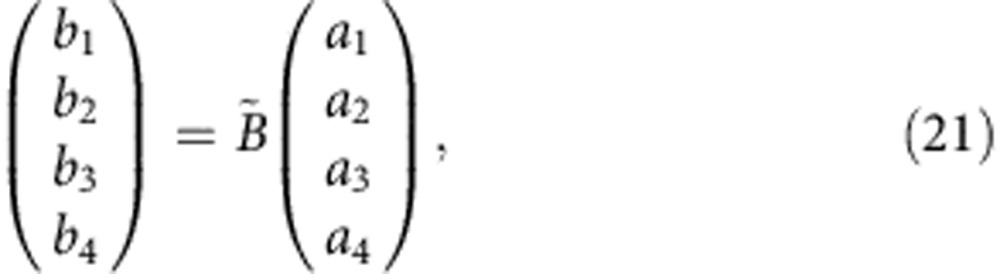


with


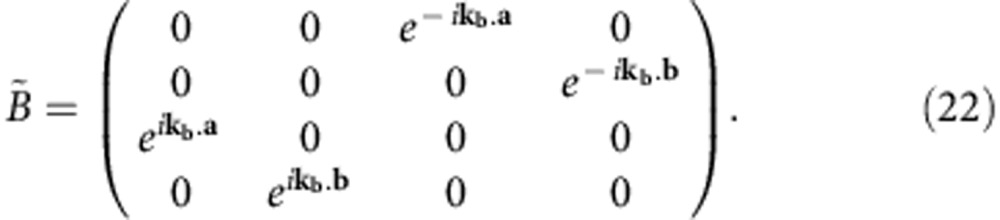


Any bulk mode must satisfy both equations (23) and (18), implying


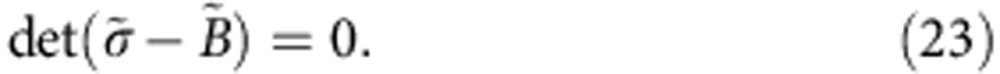


The bulk band structure can be obtained by plotting the condition number for the matrix 
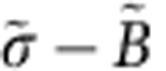
: when the condition number is infinite, 
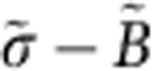
 possesses a zero eigenvalue, which is equivalent to a zero determinant. The obtained contour plots are shown in the Supplementary Fig. 3, where the evolution of the band structure of the nonreciprocal lattice is represented as a function of the Doppler bias, in perfect agreement with the numerical results presented in the main text.

### Analytical prediction of edge states

The existence of edge states can be predicted analytically by considering the scattering matrix of an array of unit cells of size 1 × 20. The first 10 unit cells are assumed to experience a right-handed Doppler bias, whereas the next 10 cells are oppositely biased, effectively creating a domain wall between the tenth and the eleventh cells. The scattering matrix of this 42 port system is calculated numerically, and as in the case of a single unit cell, periodic boundary conditions are enforced. We obtain again an equation similar to equation (24), involving a 42 by 42 determinant that needs to vanish. The result for a velocity bias of 9 m s^−1^ is shown in the Supplementary Fig. 4, in total agreement with the numerical results presented in the main text.

### Sound equations in moving media

The equations for sound propagation in moving background medium can be derived from the fluid dynamics equations by their linearization with respect to the background velocity **v** (ref. 34), which assume the form


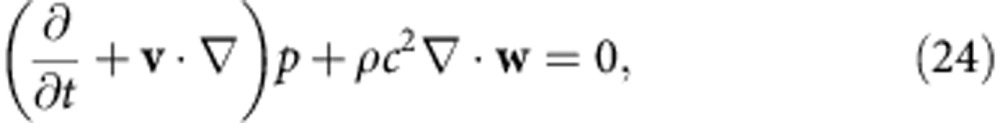



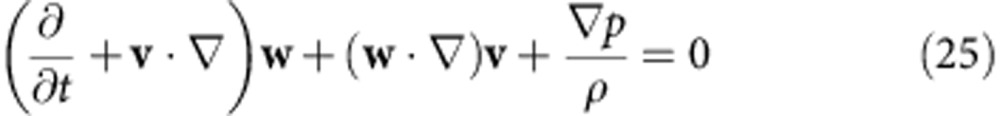


where *p* is sound pressure, **w** is particle velocity field of the sound wave, **v** is the bias velocity of the background medium supporting propagation of sound with the speed *c*, and *ρ* is its mass density. By assuming harmonic dependence of the field exp(*iωt*) one obtains an eigenvalue problem for the frequency *ω* (equation 32 of ref. 34), which was implemented as a partial differential equation model in COMSOL Multiphysics. The standard hard wall boundary conditions **n**·**w=**0 have been applied on all the boundaries of the resonators except the opening of the connectors where the Floquet periodic boundary conditions has been implemented by introducing the Bloch phase shift for the fields. The Berry curvature was then found with the standard equation Ω(**k**)=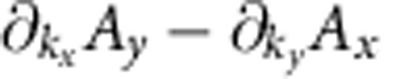, where *A*_**k**_=−*i〈p*|∂_***k***_|*p*〉 is the Berry connection, and the Chern number was calculated by integrating the Berry curvature over the Brillouin zone 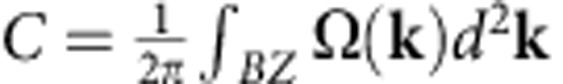.

### Multiphysics simulations of air propagation in stationary fluid flow

**Figure 6:**
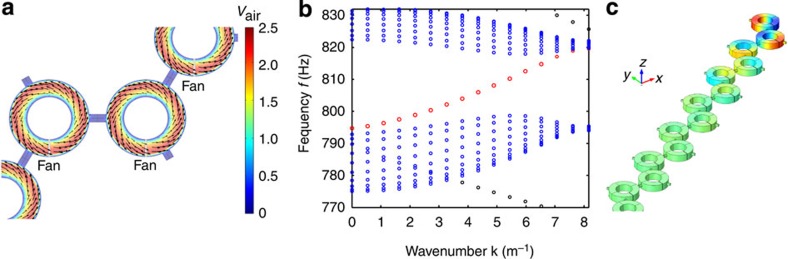
Topological edge states with mass exchange considered. (**a**)Velocity field in the supercell of the lattice found by solving the stationary Navier–Stokes equation with air motion produced by a pressure difference of 10 Pa across the fan, indicated by the grey region inside resonators. Arrows show the air velocity direction and magnitude, and the colour shows the local magnitude of the velocity, as indicated by the colour bar. (**b**) Acoustic band structure of the supercell for the velocity field shown in (**a**). Edge modes are plotted by red and black colours, and bulk modes are shown in blue. (**c**) Acoustic field profile corresponding to the topological edge mode shown by red in **b**.

To consider possible effects of nonuniformity in the velocity field that may take place in realistic structures, we have performed first-principle simulations of the airflow in the proposed periodic lattice of resonators, allowing for mass exchange between neighbouring resonators. Stationary Navier–Stokes equations were solved with the use COMSOL Multiphysics, Fluid Flow module. The periodic boundary conditions were assumed along the transverse direction of the supercell. The air motion was introduced by fans producing a pressure difference across the resonator, as shown in Fig. 6a, where the velocity field is plotted by arrows (direction and magnitude) and colour (magnitude). The obtained velocity distribution was then used to calculate the acoustic band structure and eigenmodes (Fig. 6b,c). We found that, because the velocity field is nearly the same across the connectors between resonators, the resultant pressure difference is also insignificant. As a result, the airflow across neighbouring resonators was found to be small compared to the one inside the resonators. The major difference for the velocity field calculated from the flow equations as compared to the field profile used in the main text is found in the nonuniform field profile along the transverse (radial and vertical) directions, which do not directly impact the topological order of the system. The results of the acoustic simulations using these velocity fields, shown in Fig. 6b,c, indeed demonstrate that the topological edge modes remain unaffected, confirming the inherent robustness of the proposed topological protection.

## Additional information

**How to cite this article:** Khanikaev, A. B. *et al*. Topologically robust sound propagation in an angular-momentum-biased graphene-like resonator lattice. *Nat. Commun.* 6:8260 doi: 10.1038/ncomms9260 (2015).

## Supplementary Material

Supplementary Information Supplementary Figures 1-4.
