# Supplementary material for: Topologically robust sound propagation in an angular-momentum-biased graphene-like resonator lattice
Source: Nat Commun. 2015 Oct 6;6:8260. doi: 10.1038/ncomms9260 (PMC4600716; doi:10.1038/ncomms9260)
Supplement: Supplementary Information — Supplementary Figures 1-4. [file ncomms9260-s1.pdf]

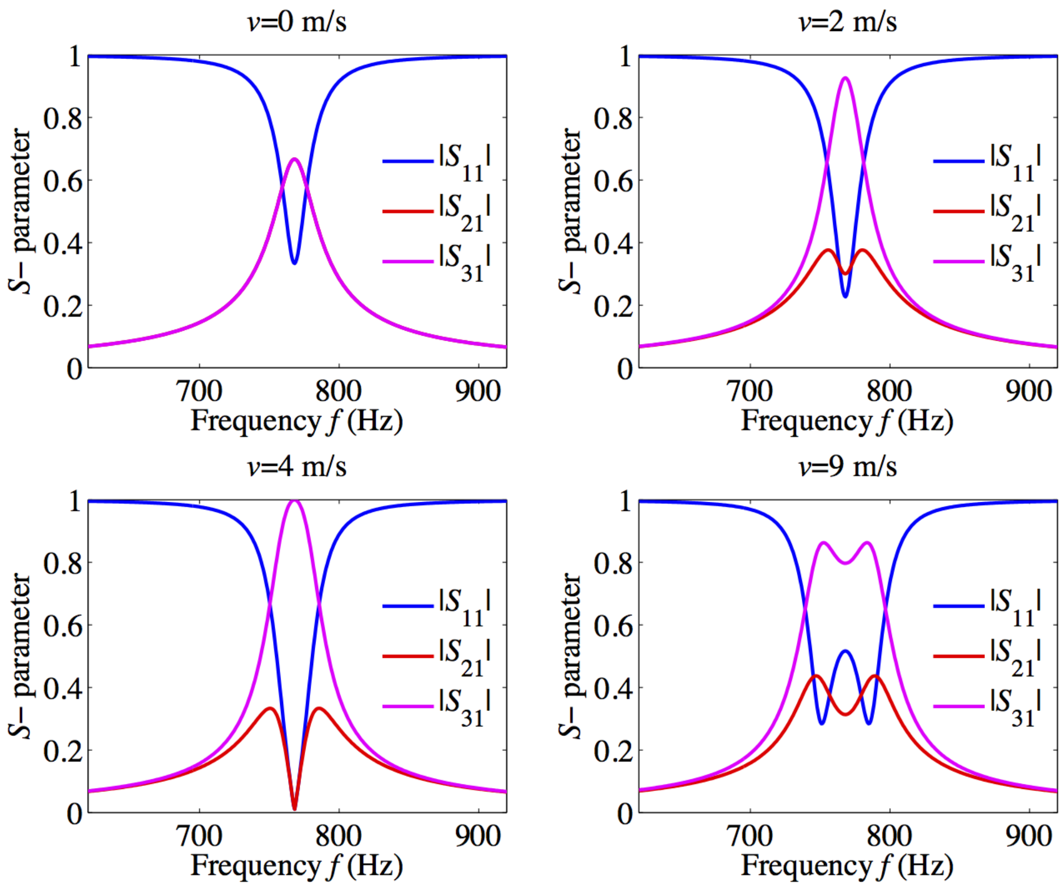

Supplementary Figure 1| Scattering parameters. Magnitude of the scattering parameters versus frequency for a single lattice element, for different values of the Doppler bias.

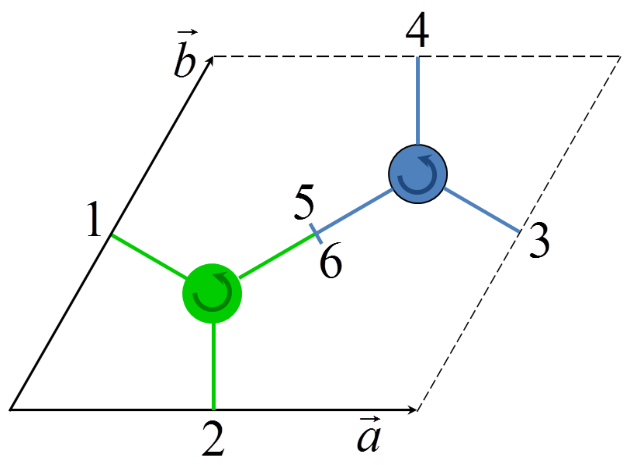

Supplementary Figure 2| Unit cell of the lattice. Unit cell schematics of the acoustic graphene, and port notations.

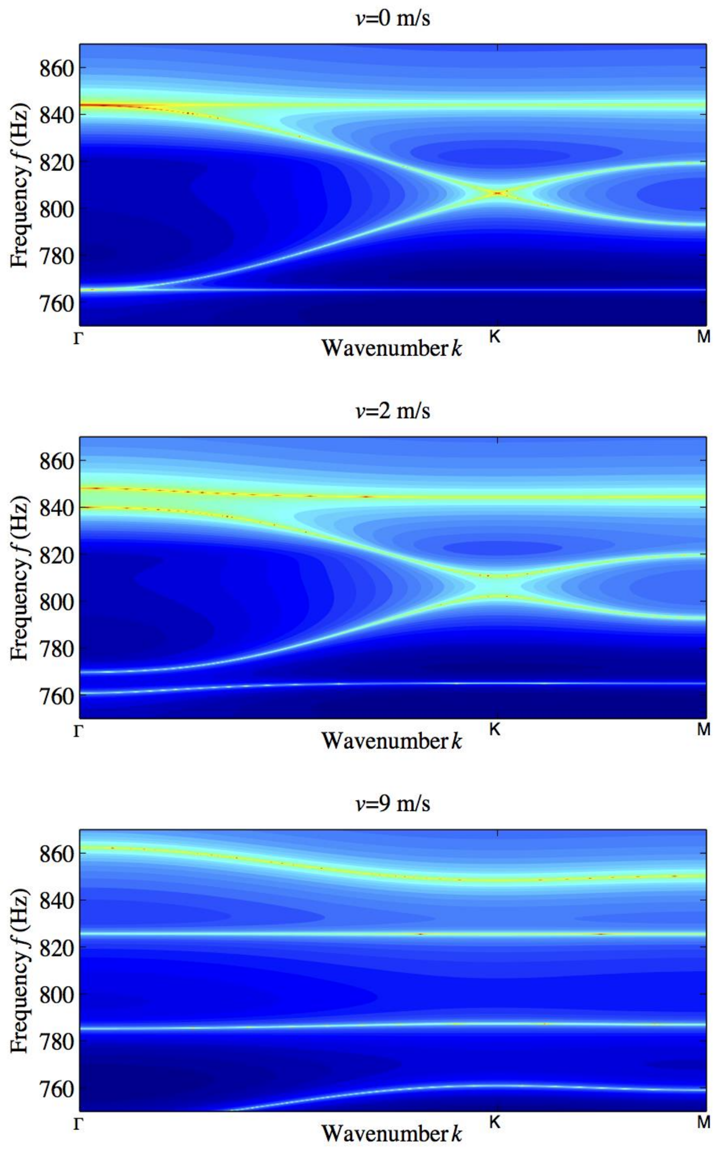

Supplementary Figure 3| Bulk band structure based on the analytical model. Analytical predictions for the bulk band structure for different values of the Doppler bias.

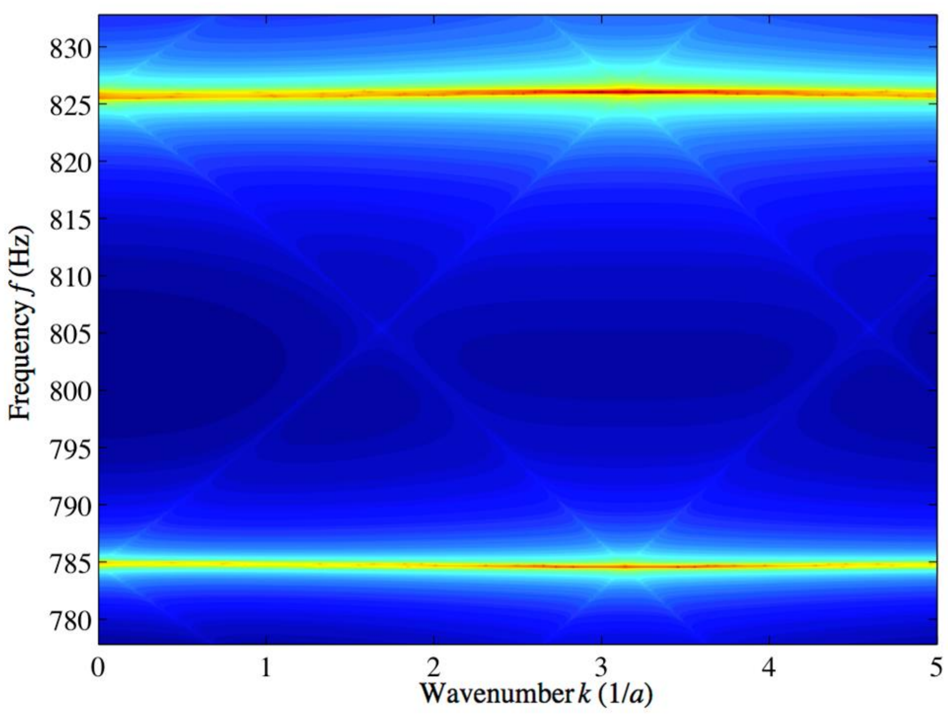

Supplementary Figure 4| Supercell band structure based on the analytical model. Analytically evaluated band structure for the supercell of 1 by 20 unit cells of acoustic graphene with the domain wall at the center. The velocity bias flips form v=9m/s to v=-9m/s along the domain wall.
